# Prosopagnosia as a Type of Conversion Disorder

**DOI:** 10.1155/2018/5972954

**Published:** 2018-02-13

**Authors:** Clodagh Power, Oisin Hannigan, Robert Coen, Irene Bruce, Matthew Gibb, Marie McCarthy, David Robinson, Brian A. Lawlor

**Affiliations:** Mercer's Institute for Successful Ageing, St. James's Hospital, Dublin, Ireland

## Abstract

**Background:**

Conversion disorder is a common and debilitating condition that remains poorly understood. We present a previously undescribed form of conversion disorder to highlight the complexity of the condition and consider the interplay of factors that produce conversion symptoms.

**Case:**

A 50-year-old male presented with acquired prosopagnosia and language impairment. Neuropsychological testing indicated right temporal lobe dysfunction. Extensive work-up outruled an organic aetiology. Reactivation of childhood trauma coincided with the onset of his symptoms. Childhood trauma is known to have adverse effects on the developing brain which may affect an individual's emotional behaviour and coping style. Functional neuroimaging techniques suggest that conversion symptoms may be linked to the disruption of higher order neural circuitry involved in the integration of emotional processing and cortical functioning.

**Conclusions:**

We propose that our patient's adverse childhood experiences led to the development of a particular personality and coping style that “primed” him for a later abnormal emotional and behavioural response when confronted with reminders of his traumatic background. Further interdisciplinary studies are required to further elucidate the neurobiological basis for this condition.

## 1. Introduction

Conversion disorder is a stigmatised and stigmatising condition that causes significant distress as well as substantial social and occupational disability. Traditionally, conversion disorder has been viewed—and often dismissed—as a “functional” phenomenon. However, research is now uncovering the neurophysiological consequences of trauma on the developing brain and the advent of functional neuroimaging techniques has the potential to allow us to explore how changes in neural circuitry may explain the origin of conversion symptoms. Though this research is in its infancy, our case report demonstrates that a formulation of conversion disorder now needs to draw from the fields of neurology, psychiatry, and developmental neuropsychology in order to understand its complex aetiology.

## 2. Case

A 50-year-old right-handed male is referred to a memory clinic with a three-year history of impaired facial recognition and language difficulties. The referring physician was concerned about a potential emerging neurodegenerative process such as Alzheimer's Disease or a temporal variant of Frontotemporal Dementia. The patient described difficulty registering and recognising faces, including his own and those of his wife and family. The first occasion that he can recall experiencing this was after his son had a hair-cut. His wife likened him to the patient's brother but he was unable to picture him. Things progressed from there. He has walked past his wife repeatedly while looking for her in a supermarket. He fails to recognise customers in the store where he works as manager despite having had significant dealings with them previously. He has difficulty identifying people in photos or recognising famous actors on TV. He uses cues such as voice recognition, tattoos, and an awareness of clothing to assist him day to day. His wife now wears a specific red coat when out together to make it easier for him to identify her. He has no trouble recalling semantic information about people once identified. When asked to visualise and describe his wife's face, he correctly described her as having brown hair but was unable to describe any additional distinguishing detail beyond the intellectual awareness that she has two eyes, a nose, and a mouth. In a similar manner, when looking at himself in the mirror, he describes being aware that he is looking at, and therefore must be seeing, his own face, rather than experiencing a feeling of recognition or familiarity. He also reported word finding difficulties in conversation and a tendency to use an inappropriate but related word inadvertently. There were no other cognitive complaints. Episodic memory, spatial and temporal orientation, navigational ability, and praxis skills were well preserved according to both subjective and collateral information.

He had no formal psychiatric history but reported long-standing anxiety symptoms. These had improved since he had stared pregabalin 75 mg twice daily for nonspecific muscular aches and pains for which a diagnosis of fibromyalgia was being considered by his GP. He was otherwise well. He was a nonsmoker and nondrinker. There was no history of seizures, headaches, or head injury. His mother had an unspecified dementia in her mid-70 s. He completed second-level education and subsequently undertook a two-year course in hotel management.

His wife described him premorbidly as an anxious personality who was quite rigid in his thinking processes and liked structure and planning. She always saw him as somewhat “emotionally detached” though she described a close and loving relationship with her and their children. She described him as an introvert who always have been prone to periods of withdrawal and quietness though these seemed to be happening more frequently in recent years.

A history of childhood sexual abuse was disclosed at a subsequent meeting. He had left his childhood home in his late teens and spent over 30 years abroad. His return to his native country coincided with emerging reports of institutional sexual abuse which were receiving widespread media coverage at the time. In retrospect, this was also the period when the first cognitive symptoms were noted by the patient.

Assessment at the memory clinic included geriatric, neurologic, psychiatric, and specialist nursing review as well as in-depth neuropsychological evaluation. Physical examination, including testing of vision, was normal. A full panel of blood showed elevated cholesterol only.

Neuropsychological testing demonstrated objective evidence of a relatively isolated prosopagnosia. See Table 1. The Northwestern University Famous Faces (NUFFACE) test consists of a series of 20 images of famous faces such as Elvis Presley and Princess Diana [1]. Only four were correctly identified by our patient. A qualitative interpretation of this test provides additional helpful information. For example, our patient was able to appreciate that there were differences between the faces but he seemed to struggle to link those differences to a memory of having seen those faces before. Another interesting observation was his use of nonfacial details to assist him. A picture of Albert Einstein was identified as an image of scientist Doc Brown from the film “Back to the Future,” likely due to the characteristic hair styles common to both. Similarly, a photo of Humphrey Bogart with his hat tilted at a jaunty angle was tentatively proposed to be a member of the Rat Pack. By contrast, Muhammad Ali triggered a feeling of recognition in our patient but it took a significant length of time (during which three other photos were discussed) before he was correctly identified. He performed poorly, also, on the Wechsler Memory Scale, 3rd edition (WMS III) Face Recognition Test which examines immediate and delayed recognition. In addition to poor performance on face recognition tests, his performance on the Trail Making Test and F-A-S test was also impaired. General memory functioning was preserved and there was no evidence of a general object agnosia as shown by perfect scores on the Word Picture Match and Pyramids and Palm Trees tests. The patient was noted to engage well and apply effort throughout and was aware of and frustrated by his failures. Though his answers were short, he was articulate with no evidence of agrammatism or hesitancy in conversation.

Brain Magnetic Resonance Imaging (MRI) was normal with no visible structural lesion and no focal atrophy. Brain Fluorodeoxyglucose-Positron Emission Tomography (FDG-PET) showed a normal pattern of FDG metabolism. Lumbar puncture for cerebrospinal fluid (CSF) biomarker analysis was negative for an Alzheimer's signature. On the basis of these results, we concluded that there was no evidence of a structural or neurodegenerative cause for his symptoms. The patient was open to considering a potential psychological basis for his complaints and agreed to a referral for psychotherapeutic evaluation.

When seen at the memory clinic for follow-up 18 months later, the patient reported no change in his symptoms, with persisting difficulty with face recognition and word finding problems. He continues to greet his wife as a customer when she visits him at work. The nonspecific muscular pain and anxiety symptoms had improved, however, on low dose amitriptyline. He had begun a programme of trauma-focussed counselling under the care of his community psychiatric team. As their work progressed, it emerged that he can recollect in clear and vivid detail the face of his abuser.

We hypothesise that this case represents an unusual form of conversion disorder. Conversion disorder commonly presents with a loss of motor or sensory function, for example, limb paralysis or blindness. However, our patient's symptoms of prosopagnosia and a specific language impairment are more unusual in that they are relatively discreet deficits which indicate dysfunction of two apparently distinct systems: facial recognition and language. At the neurobiological level, however, both are localisable to dysfunction of the right temporal lobe, suggesting a plausible location for a causative insult. As far as we can determine, an organic pathology has been outruled and we propose, therefore, a psychological basis for these symptoms which we believe have arisen as a result of a conversion-type reaction. To the best of our knowledge, this is the first such reported case of a conversion disorder presenting in this way. We suggest that it serves to highlight the degree to which abnormal emotional processing can influence and disrupt the functioning of precise brain networks.

## 3. Discussion

Conversion disorder is a syndrome of neurological symptoms of nonorganic origin often arising in the context of emotional stress. The aetiology is poorly understood. Freudian theory would suggest that our patient's symptoms have arisen as a result of repression, leading to the “conversion” of his psychological distress into physical symptoms. The association of conversion disorder with trauma has long been recognised, though it is notable that reference to a psychological basis for symptoms has been removed from the criteria for conversion disorder in Diagnostic and Statistical Manual, Fifth Edition (DSM-5) [2].

Along with dissociative disorder and somatisation disorder, conversion disorder has been recognised for almost 4,000 years under the rubric of “Hysteria,” a term that disappeared in 1980 when DSM and International Statistical Classification of Diseases and Related Health Problems (ICD) defined three distinct illnesses [3]. The substantial overlap in symptomatology between the three conditions, however, is now a source of renewed interest. Dissociative disorders may present with somatic and/or conversion symptoms, for example [4–7], and those with a somatoform disorder may also endorse dissociative experiences, particularly where there is a history of trauma [8–11]. Conceptualising these syndromes as clinical manifestations of psychoform and/or somatoform dissociation can help to explain their close phenomenological overlap. Our patient, too, described somatic symptoms in the form of vague aches and pains which were under investigation for a possible diagnosis of fibromyalgia. Unfortunately, we did not consider applying scales such as the Somatoform Dissociation Questionnaire (SDQ-20) [12] and the Dissociative Experiences Scale (DES) [13] which have been developed to objectively measure somatoform and psychoform dissociation. When applied to groups with conversion disorder, dissociative disorder, and somatisation disorder, they demonstrate the interplay of symptoms among the three conditions. Conversion disorder emerges as particularly closely affiliated with dissociative disorder, providing support for the ICD-10 classification of conversion disorder among the dissociative disorders [3, 14]. Conversion disorder remains within the Somatic Symptom and Related Disorders category in DSM-5 [2]. Systematic assessment of psychiatric patients for dissociative disorders has shown that these conditions are far more prevalent than is routinely identified with significantly higher rates of childhood trauma seen than among those who do not endorse dissociative symptoms [15, 16].

A precise neurobiological explanation for conversion disorder remains elusive though it is known that early life stress can result in maladaptive alterations to brain circuitry and neurochemistry. Preliminary evidence indicates that basal cortisol levels are elevated among individuals who experience psychogenic nonepileptic seizures (PNES) and this seems to correlate with levels of childhood abuse [17]. The prefrontal cortex, an area important for self-regulatory and goal-directed behaviours, appears particularly vulnerable to the effects of stress-induced changes of the hypothalamic-pituitary-adrenal (HPA) axis [18]. Certain aspects of our patient's personality and coping style would certainly fit with this hypothesis, though it fails to explain the late onset of distinct cognitive symptoms.

A recent review describes the outcomes of studies using functional neuroimaging to characterise the brain areas considered pertinent to the generation of conversion symptoms [19]. Importantly, results differed between those with conversion disorder, those feigning symptoms, and healthy controls. Disruption to the regulating processes of the higher order frontal networks was a common finding in those with conversion symptoms. The small number of studies which considered nonmotor conversion disorder showed that, in general, functional MRI activity was reduced in primary sensory areas but increased in other areas such as the frontal cortices and subcortical nuclei, areas which are important for emotional processing, regulation, and response as well as the integration of information across brain domains.

Our patient is notable in that his two presenting complaints—impairment of face recognition and language—point to dysfunction of a relatively precise neuroanatomical area. Though face recognition and identification requires the integration of information from an extended network that includes the orbitofrontal cortex, inferior frontal gyrus, posterior cingulate, and inferior occipital gyri, acquired prosopagnosia may be localised to disruption of the fusiform face area (temporal lobe) and the superior temporal sulcus [20]. The temporal lobes are central, also, to language, though the neuroanatomical area responsible for language generation and comprehension is also diffuse. Impairment of the F-A-S test indicates a more anterior, executive-type deficit as does impaired performance on the Trail Making Test. While face processing is more active in the right hemisphere and word processing more so in the left, both show bilateral networks that overlap and there are some small studies to suggest that prosopagnosics may have impairments of word processing, though results are inconsistent and difficult to interpret [21]. Given the intimacy of neural connectivity in this region, however, it is not implausible to consider that a relatively subtle insult may impact on multiple cognitive functions.

In the case of our patient, we hypothesise that the effects on his developing brain of his adverse childhood experiences “primed” him for an abnormal neuropsychological response upon exposure to reminders of his traumatic past. We propose that the degree of repressed trauma that surfaced resulted in dysregulation of his higher order frontal and subcortical emotional processing systems and their integration with the functioning of his right temporal lobe.

The Structural Theory of Dissociation conceptualises this elegantly from a developmental psychology perspective. It sets out how childhood trauma compromises the successful integration of the varying parts of the developing self with consequences for adult behaviours and psychosocial interactions [22, 23]. Drawing from evolutionary psychology, learning theory and attachment theory, it describes how severe trauma in childhood results in a structural dissociation of the personality into a part that maintains everyday functioning and is phobically avoidant of the trauma memories (the “apparently normal” part, or ANP) and a part that remains immersed in the trauma and is responsive to a narrow range of threat-oriented cues (the “emotional” part or EP) [24, 25]. Dissociation in this context may be simultaneously regarded as both a defence mechanism, in that it allows attention to the matters of everyday life, and a deficit, in that these disparate parts fail to integrate into a cohesively functioning personality. In the case of our patient, who fled childhood institutional abuse and disrupted attachments, the apparently normal part permitted successful educational attainment, occupational functioning, and a happy marital and family life while the emotional part was the source of the memory of the perpetrator, guilt, shame, stress, and defence against threat.

Factors that are thought to indicate a better prognosis in conversion disorder are an acceptance of a psychological aetiology, sudden onset and short duration of symptoms, an identifiable emotional stressor, no ongoing litigation, good premorbid functioning, and no comorbid psychiatric illness [26]. On this basis, our patient's prognosis is potentially quite good. He is engaging with psychotherapy to explore the emotional basis of his symptoms and has responded well to the treatment of his anxiety. In psychological terms, resolution of the emotional conflict should result in resolution of his symptoms.

He is displaying remarkable resilience in coping with the day to day difficulties associated with prosopagnosia. There is evidence to demonstrate that prosopagnosics may struggle with interpersonal relations, tend to avoid social interactions, are disadvantaged in their careers, suffer from poor self-confidence, and experience anxiety about inadvertently offending others [27–29]. Most of the work pertaining to the psychosocial consequences of prosopagnosia has been carried out among those with developmental prosopagnosia (DP), where the deficit is present from birth and often diagnosed quite late (or not at all) in part because of the variety of coping skills which are developed, embarrassment about a potentially socially disabling impairment, or, simply, a lack of awareness of an impairment in a specific skill that the majority of the population take for granted [30]. Our patient's use of nonfacial cues such as clothing and voice recognition are common compensatory mechanisms employed by prosopagnosics [29, 31]. Our patient has had the benefit of premorbidly established robust social networks and a supportive place of work. On the other hand, he has had to hone these coping strategies, de novo, within a relatively short period.

Much remains to be learned about conversion disorder and the interplay of factors that give rise to symptoms. One of the most intriguing aspects of this condition is that it crosses the traditionally well-defined boundaries of physical and psychological illness. It challenges us to integrate our understanding of neurology, endocrinology, and immunology with developmental psychology, neuropsychology, and general psychiatry and neuropsychiatry. The use of functional neuroimaging in this field is in its infancy but holds significant promise. A major challenge is to identify groups of patients with sufficiently homogenous symptomatology to allow meaningful interpretation of the findings as it is likely that the affected neurocircuitry of a patient suffering with limb paralysis, for example, will differ from that of a person experiencing PNES. To date, there have been no neuroimaging studies to explore the neurobiological effects of the treatment of conversion disorder. What happens at a neurophysiological level in the brains of those whose symptoms resolve following successful treatment? And is there an identifiable difference from those who are treatment resistant or whose symptoms resolve spontaneously? The stress-diathesis model [32] is a helpful framework to explain why some individuals are seemingly more vulnerable to stressful events than others but large, multidisciplinary, longitudinal studies that follow subjects rigorously from childhood would be necessary to definitively explore whether the model is actually valid in this clinical scenario. There would be other benefits to a study of this ambition, however, such as an opportunity to objectively compare how different individuals cope with adversity over time and to explore the role of genetics and environment. Ultimately, a more nuanced understanding of this debilitating and stigmatising condition will require a cross-disciplinary collaboration that integrates the expert knowledge of all stakeholders.

## Figures and Tables

**Table 1:** Results of neuropsychological evaluation at memory clinic assessments 1 and 2 (18-month interval between visits).

Test	Assessment 1	Assessment 2
ACE-R	85/100	87/100
Delayed Word Recall	Free recall 4/10 Delayed recall 10/10	Not repeated
Boston Naming Test	30th % ile	Not repeated
Animal fluency	50th % ile	12th % ile
Letter fluency	<1st % ile	2nd % ile
Word Picture Match	48/48	Not repeated
Pyramids and Palm Trees	52/52	Not repeated
WAIS III Vocabulary	37th % ile	Not repeated
Trail Making	Part A: <1st % ile Part B: 8th % ile	Part A: 5th % ile Part B: 4th % ile
Rey Osterrieth Complex Figure	Normal for copy and retention after delay	Not repeated
NUFACE Test	4/20	9/20
WMS III Face Recognition	Immediate recognition: 9th % ile Delayed recognition: 5th % ile	Immediate: 16th % ile Delayed: 16th % ile
